# Tris(phenanthroline-κ^2^ *N*,*N*′)cobalt(II) tetra­fluoridoborate acetonitrile solvate

**DOI:** 10.1107/S1600536808036611

**Published:** 2008-11-13

**Authors:** David J. Harding, Phimphaka Harding, Harry Adams

**Affiliations:** a Molecular Technology Unit Cell, Department of Chemistry, Walailak University, Thasala, Nakorn Si Thammarat 80161, Thailand; b Department of Chemistry, Faculty of Science, University of Sheffield, Brook Hill, Sheffield S3 7HF, England

## Abstract

In the crystal structure of the title compound, [Co(C_12_H_8_N_2_)_3_](BF_4_)_2_·CH_3_CN, the mol­ecular packing involves dimers of distorted octahedrally coordinated cations which are held together by one π–π [centroid–centroid = 3.542 (4) Å] and two C—H⋯π inter­actions  [2.573 (4) Å] resulting in a P4AE (Parallel Fourfold Aryl Embrace) motif. The anions are found in aryl boxes formed from the phenanthroline ligands.

## Related literature

For other [Co(phen)_3_]^2+^ complexes, see: Boys *et al.* (1984 ▶); Geraghty *et al.* (1999 ▶); Russell *et al.* (2001 ▶); Tershansy *et al.* (2005 ▶).
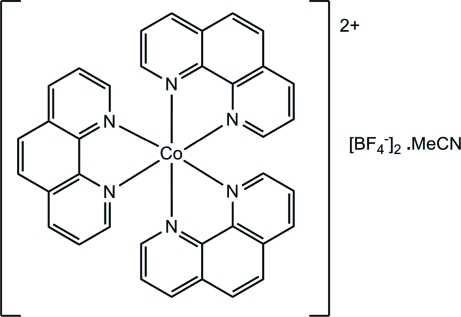


## Experimental

### 

#### Crystal data

[Co(C_12_H_8_N_2_)_3_](BF_4_)_2_·C_2_H_3_N
*M* *_r_* = 814.22
Monoclinic, 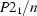

*a* = 18.0443 (2) Å
*b* = 9.36230 (10) Å
*c* = 22.0702 (2) Å
β = 107.3610 (10)°
*V* = 3558.60 (6) Å^3^
*Z* = 4
Mo *K*α radiation
μ = 0.56 mm^−1^
*T* = 150 (2) K
0.32 × 0.28 × 0.12 mm

#### Data collection

Bruker SMART CCD area-detector diffractometer
Absorption correction: multi-scan (*SADABS*; Bruker, 1997 ▶) *T*_min_ = 0.840, *T*_max_ = 0.935
56541 measured reflections
6276 independent reflections
5268 reflections with *I* > 2σ(*I*)
*R*_int_ = 0.046

#### Refinement

*R*[*F* ^2^ > 2σ(*F* ^2^)] = 0.072
*wR*(*F* ^2^) = 0.213
*S* = 1.03
6276 reflections
501 parameters
H-atom parameters constrained
Δρ_max_ = 2.96 e Å^−3^
Δρ_min_ = −0.94 e Å^−3^

### 

Data collection: *SMART* (Bruker, 1997 ▶); cell refinement: *SAINT* (Bruker, 1997 ▶); data reduction: *SAINT*; program(s) used to solve structure: *SHELXS97* (Sheldrick, 2008 ▶); program(s) used to refine structure: *SHELXL97* (Sheldrick, 2008 ▶); molecular graphics: *SHELXTL* (Sheldrick, 2008 ▶); software used to prepare material for publication: *SHELXTL*.

## Supplementary Material

Crystal structure: contains datablocks I, global. DOI: 10.1107/S1600536808036611/at2665sup1.cif

Structure factors: contains datablocks I. DOI: 10.1107/S1600536808036611/at2665Isup2.hkl

Additional supplementary materials:  crystallographic information; 3D view; checkCIF report

## Figures and Tables

**Table 1:** Selected geometric parameters (Å, °)

Co1—N4	2.123 (4)
Co1—N2	2.129 (4)
Co1—N6	2.129 (4)
Co1—N1	2.131 (3)
Co1—N5	2.133 (4)
Co1—N3	2.142 (4)

**Fig. 1.**
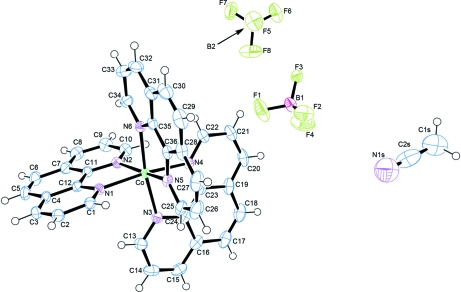
The molecular structure of (1) showing the atom-labelling scheme. Displacement ellipsoids are drawn at the 50% probability level.

**Fig. 2.**
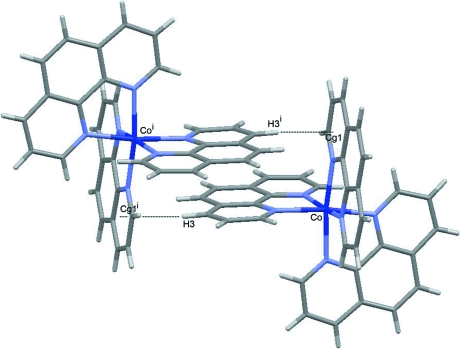
The molecular packing in (1) showing the C—H···π and π–π interactions which make up the P4AE structural motif. Only selected H atoms are labelled for clarity. [Symmetry codes:(i) -x, -y, 1 - z].

## supplementary crystallographic information

### Comment

The reaction of anhydrous cobalt(II) chloride with AgBF_4_ in the presence of phenanthroline yields the coordination compound tris(phenanthroline)cobalt(II) tetrafluoroborate (1), [Co(phen)_3_][BF_4_]_2_.MeCN. Crystals were grown by allowing ether to diffuse into a concentrated solution of the complex in MeCN. The title complex crystallizes in the space group P2_1_/n in contrast to the related compound [Co(phen)_3_][BF_4_]_2_.H_2_O.EtOH which crystallizes in P1 (Russell *et al.*, 2001). The structure of (1) is shown in Fig. 1 while important bond lengths and angles are given in Table 1. The cobalt centre is octahedrally coordinated with Co—N bond lengths and N—Co—N angles for the chelating phenanthroline ligands essentially identical to those reported for other [Co(phen)_3_]^2+^ complexes (Boys *et al.*, 1984; Geraghty *et al.*, 1999; Russell *et al.*, 2001; Tershansy *et al.*, 2005).

The crystal lattice of (1) contains dimers of [Co(phen)_3_]^2+^ cations in which there is a P4AE (Parallel Fourfold Aryl Embrace) motif involving one π–π [centroid···centroid 3.542 (4) Å] and two C—H···π interactions between the phenanthroline ligands as shown in Fig. 2 (Cg1 is the centroid of the ring C31–C35; Russell *et al.*, 2001). The offset between the central aryl ring of the two phenanthroline ligands is 6.443 (3) Å and indicative of overlap of a single aryl ring (Russell *et al.*, 2001). The dimers found in (1) are isolated from each other unlike in the structure of [Co(phen)_3_][BF_4_]_2_.H_2_O.EtOH where a further P4AE interaction results in formation of a zig-zag chain. A further difference is that the anions are not found in hydrophilic channels between chains of the cations but rather in aryl boxes formed from six phenanthroline ligands. This difference is presumeably the result of a lack of suitable hydrogen bonding solvent in the current structure.

### Experimental

Cobalt(II) chloride (130 mg, 1 mmol) was suspended in MeCN (20 ml). AgBF_4_ (389 mg, 2 mmol) was then added resulting in precipitation of a white solid. The solution was filtered through celite to remove AgCl and phenanthroline (541 mg, 3 mmol) was added giving an orange solution. The volume of the solution was reduced in vacuo to *ca.* 10 ml and then layered with Et_2_O (60 ml). After two days yellow crystals formed (602 mg, 74%) Analysis calculated for C_38_H_27_N_7_B_2_F_8_Co: C 56.06, H 3.34, N 12.04%; found: C 56.27, H 3.40, N 12.41%. ESI^+^ MS: (*m/z*) Anal. Calc. 814.22; found: [M]^+^ 814.19.

### Refinement

Hydrogen atoms were placed geometrically and refined with a riding model and with *U*_iso_ constrained to be 1.2 (aromatic CH) or 1.5 (Me) times *U*_eq_ of the carrier atom.

### Crystal data

**Table:**

[Co(C_12_H_8_N_2_)_3_](BF_4_)_2_·C_2_H_3_N	*F*_000_ = 1652
*M**_r_* = 814.22	*D*_x_ = 1.520 Mg m^−^^3^
Monoclinic, *P*2_1_/*n*	Mo *K*α radiation λ = 0.71073 Å
Hall symbol: -P 2yn	Cell parameters from 9965 reflections
*a* = 18.0443 (2) Å	θ = 2.4–24.8º
*b* = 9.36230 (10) Å	µ = 0.57 mm^−^^1^
*c* = 22.0702 (2) Å	*T* = 150 (2) K
β = 107.3610 (10)º	Plate, yellow
*V* = 3558.60 (6) Å^3^	0.32 × 0.28 × 0.12 mm
*Z* = 4	

### Data collection

**Table:**

Bruker SMART CCD area-detector diffractometer	6276 independent reflections
Radiation source: fine-focus sealed tube	5268 reflections with *I* > 2σ(*I*)
Monochromator: graphite	*R*_int_ = 0.046
Detector resolution: 100 pixels mm^-1^	θ_max_ = 25.0º
*T* = 150(2) K	θ_min_ = 1.3º
φ and ω scans	*h* = −21→21
Absorption correction: multi-scan(SADABS; Bruker, 1997)	*k* = −11→11
*T*_min_ = 0.840, *T*_max_ = 0.935	*l* = −26→26
56541 measured reflections	

### Refinement

**Table:**

Refinement on *F*^2^	Secondary atom site location: difference Fourier map
Least-squares matrix: full	Hydrogen site location: inferred from neighbouring sites
*R*[*F*^2^ > 2σ(*F*^2^)] = 0.072	H-atom parameters constrained
*wR*(*F*^2^) = 0.213	*w* = 1/[σ^2^(*F*_o_^2^) + (0.1194*P*)^2^ + 14.1582*P*] where *P* = (*F*_o_^2^ + 2*F*_c_^2^)/3
*S* = 1.03	(Δ/σ)_max_ < 0.001
6276 reflections	Δρ_max_ = 2.96 e Å^−^^3^
501 parameters	Δρ_min_ = −0.94 e Å^−^^3^
Primary atom site location: structure-invariant direct methods	Extinction correction: none

### Special details

**Table:**

Geometry. All e.s.d.'s (except the e.s.d. in the dihedral angle between two l.s. planes)are estimated using the full covariance matrix. The cell e.s.d.'s are takeninto account individually in the estimation of e.s.d.'s in distances, anglesand torsion angles; correlations between e.s.d.'s in cell parameters are onlyused when they are defined by crystal symmetry. An approximate (isotropic)treatment of cell e.s.d.'s is used for estimating e.s.d.'s involving l.s. planes.
Refinement. Refinement of *F*^2^ against ALL reflections. The weighted *R*-factor *wR* andgoodness of fit *S* are based on *F*^2^, conventional *R*-factors *R* are basedon *F*, with *F* set to zero for negative *F*^2^. The threshold expression of*F*^2^ > σ(*F*^2^) is used only for calculating *R*-factors(gt) *etc*. and isnot relevant to the choice of reflections for refinement. *R*-factors basedon *F*^2^ are statistically about twice as large as those based on *F*, and *R*-factors based on ALL data will be even larger.

### Fractional atomic coordinates and isotropic or equivalent isotropic displacement parameters (Å^2^)

**Table:**

	*x*	*y*	*z*	*U*_iso_*/*U*_eq_	
Co1	0.22845 (3)	0.04969 (6)	0.47231 (3)	0.0198 (2)	
N1	0.1221 (2)	−0.0494 (4)	0.47236 (17)	0.0223 (8)	
N2	0.1549 (2)	0.1052 (4)	0.38050 (17)	0.0233 (8)	
N3	0.2689 (2)	−0.1362 (4)	0.43523 (17)	0.0235 (8)	
N4	0.3331 (2)	0.1259 (4)	0.45860 (17)	0.0232 (8)	
N5	0.2832 (2)	0.0047 (4)	0.57022 (17)	0.0235 (8)	
N6	0.20568 (19)	0.2398 (4)	0.51664 (17)	0.0216 (8)	
C1	0.1066 (3)	−0.1261 (5)	0.5179 (2)	0.0252 (9)	
H1	0.1448	−0.1343	0.5568	0.030*	
C2	0.0356 (3)	−0.1949 (5)	0.5099 (2)	0.0289 (10)	
H2	0.0267	−0.2474	0.5428	0.035*	
C3	−0.0204 (3)	−0.1836 (5)	0.4530 (2)	0.0313 (11)	
H3	−0.0675	−0.2307	0.4465	0.038*	
C4	−0.0075 (2)	−0.1013 (5)	0.4041 (2)	0.0278 (10)	
C5	−0.0641 (3)	−0.0811 (6)	0.3436 (2)	0.0339 (11)	
H5	−0.1121	−0.1258	0.3351	0.041*	
C6	−0.0489 (3)	0.0016 (6)	0.2988 (2)	0.0365 (12)	
H6	−0.0872	0.0154	0.2604	0.044*	
C7	0.0253 (3)	0.0685 (5)	0.3093 (2)	0.0289 (10)	
C8	0.0446 (3)	0.1556 (6)	0.2640 (2)	0.0348 (11)	
H8	0.0081	0.1739	0.2250	0.042*	
C9	0.1171 (3)	0.2124 (6)	0.2777 (2)	0.0349 (11)	
H9	0.1303	0.2701	0.2482	0.042*	
C10	0.1712 (3)	0.1839 (5)	0.3359 (2)	0.0290 (10)	
H10	0.2210	0.2213	0.3440	0.035*	
C11	0.0829 (2)	0.0478 (5)	0.3671 (2)	0.0237 (9)	
C12	0.0656 (2)	−0.0368 (5)	0.4164 (2)	0.0234 (9)	
C13	0.2350 (3)	−0.2627 (5)	0.4210 (2)	0.0312 (10)	
H13	0.1877	−0.2785	0.4288	0.037*	
C14	0.2679 (3)	−0.3736 (5)	0.3948 (2)	0.0379 (12)	
H14	0.2419	−0.4600	0.3843	0.046*	
C15	0.3377 (3)	−0.3543 (6)	0.3847 (2)	0.0375 (12)	
H15	0.3605	−0.4282	0.3685	0.045*	
C16	0.3749 (3)	−0.2222 (5)	0.3990 (2)	0.0319 (11)	
C17	0.4484 (3)	−0.1899 (7)	0.3889 (2)	0.0408 (13)	
H17	0.4745	−0.2613	0.3742	0.049*	
C18	0.4802 (3)	−0.0585 (7)	0.4003 (3)	0.0430 (14)	
H18	0.5273	−0.0409	0.3927	0.052*	
C19	0.4429 (3)	0.0541 (6)	0.4237 (2)	0.0331 (11)	
C20	0.4724 (3)	0.1939 (6)	0.4353 (2)	0.0393 (12)	
H20	0.5186	0.2180	0.4274	0.047*	
C21	0.4328 (3)	0.2934 (6)	0.4580 (2)	0.0374 (12)	
H21	0.4521	0.3859	0.4659	0.045*	
C22	0.3631 (3)	0.2568 (5)	0.4695 (2)	0.0288 (10)	
H22	0.3368	0.3261	0.4852	0.035*	
C23	0.3720 (2)	0.0255 (5)	0.4363 (2)	0.0242 (9)	
C24	0.3377 (2)	−0.1148 (5)	0.42359 (19)	0.0238 (9)	
C25	0.3210 (3)	−0.1131 (5)	0.5963 (2)	0.0315 (10)	
H25	0.3253	−0.1884	0.5701	0.038*	
C26	0.3545 (3)	−0.1271 (7)	0.6623 (3)	0.0437 (14)	
H26	0.3812	−0.2099	0.6792	0.052*	
C27	0.3477 (3)	−0.0184 (7)	0.7013 (3)	0.0437 (14)	
H27	0.3696	−0.0269	0.7450	0.052*	
C28	0.3075 (3)	0.1062 (6)	0.6756 (2)	0.0333 (11)	
C29	0.2966 (3)	0.2253 (6)	0.7135 (2)	0.0405 (13)	
H29	0.3175	0.2215	0.7574	0.049*	
C30	0.2569 (3)	0.3414 (6)	0.6863 (2)	0.0383 (12)	
H30	0.2506	0.4167	0.7118	0.046*	
C31	0.2242 (3)	0.3520 (5)	0.6191 (2)	0.0301 (10)	
C32	0.1828 (3)	0.4708 (5)	0.5884 (3)	0.0370 (12)	
H32	0.1749	0.5486	0.6120	0.044*	
C33	0.1537 (3)	0.4724 (5)	0.5234 (3)	0.0369 (12)	
H33	0.1258	0.5507	0.5025	0.044*	
C34	0.1667 (3)	0.3550 (5)	0.4893 (2)	0.0271 (10)	
H34	0.1471	0.3572	0.4452	0.033*	
C35	0.2344 (2)	0.2377 (5)	0.5808 (2)	0.0235 (9)	
C36	0.2759 (2)	0.1130 (5)	0.6095 (2)	0.0244 (9)	
B1	0.5917 (3)	0.4996 (7)	0.3923 (3)	0.0366 (13)	
B2	0.4925 (4)	1.0025 (7)	0.1490 (4)	0.0475 (17)	
F1	0.5174 (3)	0.4621 (5)	0.3646 (2)	0.0922 (17)	
F2	0.6209 (3)	0.4429 (4)	0.45093 (17)	0.0689 (12)	
F3	0.60229 (15)	0.6448 (3)	0.39024 (15)	0.0399 (7)	
F4	0.6351 (3)	0.4370 (5)	0.3547 (2)	0.0865 (15)	
F5	0.4886 (3)	0.9846 (5)	0.2141 (2)	0.0738 (12)	
F6	0.5557 (2)	1.0845 (4)	0.15558 (16)	0.0625 (11)	
F7	0.4259 (2)	1.0742 (5)	0.1195 (2)	0.0742 (13)	
F8	0.4948 (3)	0.8703 (4)	0.1273 (2)	0.0852 (14)	
C1S	0.9015 (5)	0.2643 (9)	0.7541 (3)	0.079 (2)	
H1S1	0.8986	0.3610	0.7677	0.118*	
H1S2	0.9017	0.2640	0.7106	0.118*	
H1S3	0.9482	0.2205	0.7802	0.118*	
C2S	0.8353 (5)	0.1856 (8)	0.7595 (4)	0.072 (2)	
N1S	0.7831 (5)	0.1217 (10)	0.7631 (4)	0.098 (2)*	

### Atomic displacement parameters (Å^2^)

**Table:**

	*U*^11^	*U*^22^	*U*^33^	*U*^12^	*U*^13^	*U*^23^
Co1	0.0168 (3)	0.0217 (3)	0.0215 (3)	−0.0008 (2)	0.0067 (2)	−0.0004 (2)
N1	0.0199 (17)	0.0226 (18)	0.0255 (19)	−0.0003 (14)	0.0086 (15)	−0.0029 (14)
N2	0.0244 (18)	0.0242 (18)	0.0228 (19)	−0.0015 (15)	0.0094 (15)	−0.0027 (15)
N3	0.0224 (18)	0.0256 (19)	0.0237 (19)	0.0007 (15)	0.0086 (15)	−0.0003 (15)
N4	0.0196 (17)	0.028 (2)	0.0206 (18)	−0.0017 (15)	0.0048 (14)	0.0022 (15)
N5	0.0180 (17)	0.0275 (19)	0.0243 (19)	0.0005 (15)	0.0055 (14)	0.0053 (15)
N6	0.0198 (17)	0.0225 (18)	0.0239 (19)	−0.0019 (14)	0.0085 (14)	−0.0003 (14)
C1	0.024 (2)	0.025 (2)	0.029 (2)	0.0014 (18)	0.0118 (18)	0.0010 (18)
C2	0.030 (2)	0.027 (2)	0.037 (3)	−0.0012 (19)	0.020 (2)	−0.0002 (19)
C3	0.023 (2)	0.026 (2)	0.049 (3)	−0.0038 (18)	0.016 (2)	−0.005 (2)
C4	0.019 (2)	0.029 (2)	0.038 (3)	0.0007 (18)	0.0127 (19)	−0.008 (2)
C5	0.019 (2)	0.040 (3)	0.039 (3)	−0.001 (2)	0.003 (2)	−0.008 (2)
C6	0.025 (2)	0.041 (3)	0.036 (3)	0.001 (2)	−0.003 (2)	−0.007 (2)
C7	0.027 (2)	0.031 (2)	0.027 (2)	0.0050 (19)	0.0053 (19)	−0.0032 (19)
C8	0.037 (3)	0.040 (3)	0.023 (2)	0.005 (2)	0.003 (2)	−0.002 (2)
C9	0.042 (3)	0.039 (3)	0.024 (2)	−0.001 (2)	0.012 (2)	0.002 (2)
C10	0.031 (2)	0.031 (2)	0.026 (2)	−0.003 (2)	0.0093 (19)	−0.0006 (19)
C11	0.021 (2)	0.024 (2)	0.025 (2)	0.0012 (17)	0.0064 (18)	−0.0034 (17)
C12	0.020 (2)	0.024 (2)	0.027 (2)	0.0009 (17)	0.0081 (18)	−0.0053 (17)
C13	0.035 (3)	0.027 (2)	0.034 (3)	−0.001 (2)	0.013 (2)	−0.0016 (19)
C14	0.052 (3)	0.025 (2)	0.034 (3)	0.002 (2)	0.009 (2)	−0.004 (2)
C15	0.046 (3)	0.037 (3)	0.032 (3)	0.015 (2)	0.016 (2)	−0.002 (2)
C16	0.033 (2)	0.041 (3)	0.021 (2)	0.012 (2)	0.0076 (19)	0.001 (2)
C17	0.030 (3)	0.066 (4)	0.030 (3)	0.012 (3)	0.014 (2)	−0.006 (2)
C18	0.027 (2)	0.073 (4)	0.035 (3)	0.003 (3)	0.019 (2)	−0.004 (3)
C19	0.022 (2)	0.056 (3)	0.022 (2)	−0.002 (2)	0.0085 (19)	0.005 (2)
C20	0.024 (2)	0.062 (3)	0.033 (3)	−0.015 (2)	0.011 (2)	0.004 (2)
C21	0.033 (3)	0.042 (3)	0.034 (3)	−0.016 (2)	0.006 (2)	0.002 (2)
C22	0.028 (2)	0.031 (2)	0.025 (2)	−0.0075 (19)	0.0041 (18)	0.0014 (19)
C23	0.020 (2)	0.035 (2)	0.016 (2)	0.0003 (18)	0.0035 (16)	0.0008 (17)
C24	0.024 (2)	0.032 (2)	0.015 (2)	0.0033 (18)	0.0057 (16)	0.0008 (17)
C25	0.023 (2)	0.036 (3)	0.036 (3)	0.004 (2)	0.010 (2)	0.008 (2)
C26	0.031 (3)	0.056 (3)	0.043 (3)	0.009 (2)	0.007 (2)	0.023 (3)
C27	0.030 (3)	0.068 (4)	0.030 (3)	0.002 (3)	0.004 (2)	0.016 (3)
C28	0.024 (2)	0.052 (3)	0.022 (2)	−0.008 (2)	0.0049 (19)	0.006 (2)
C29	0.040 (3)	0.064 (4)	0.018 (2)	−0.014 (3)	0.009 (2)	−0.007 (2)
C30	0.040 (3)	0.045 (3)	0.034 (3)	−0.011 (2)	0.017 (2)	−0.014 (2)
C31	0.029 (2)	0.034 (3)	0.032 (3)	−0.010 (2)	0.015 (2)	−0.011 (2)
C32	0.042 (3)	0.026 (2)	0.051 (3)	−0.005 (2)	0.026 (3)	−0.012 (2)
C33	0.045 (3)	0.027 (3)	0.045 (3)	0.004 (2)	0.023 (2)	0.001 (2)
C34	0.027 (2)	0.027 (2)	0.030 (2)	0.0017 (18)	0.0120 (19)	0.0040 (18)
C35	0.018 (2)	0.028 (2)	0.026 (2)	−0.0055 (17)	0.0089 (17)	−0.0021 (18)
C36	0.0172 (19)	0.033 (2)	0.023 (2)	−0.0053 (18)	0.0060 (17)	0.0001 (18)
B1	0.035 (3)	0.042 (3)	0.032 (3)	−0.010 (3)	0.008 (2)	0.001 (3)
B2	0.034 (3)	0.035 (3)	0.061 (4)	−0.006 (3)	−0.004 (3)	0.006 (3)
F1	0.066 (3)	0.086 (3)	0.096 (3)	−0.043 (2)	−0.020 (2)	0.043 (3)
F2	0.096 (3)	0.051 (2)	0.040 (2)	0.005 (2)	−0.0088 (19)	0.0005 (16)
F3	0.0271 (14)	0.0351 (16)	0.060 (2)	−0.0011 (12)	0.0168 (13)	0.0045 (14)
F4	0.129 (4)	0.062 (3)	0.089 (3)	−0.011 (3)	0.066 (3)	−0.017 (2)
F5	0.078 (3)	0.089 (3)	0.059 (2)	−0.013 (2)	0.027 (2)	0.001 (2)
F6	0.055 (2)	0.075 (2)	0.043 (2)	−0.0238 (18)	−0.0066 (16)	0.0258 (18)
F7	0.0396 (19)	0.105 (3)	0.069 (3)	0.012 (2)	0.0018 (18)	−0.033 (2)
F8	0.132 (4)	0.048 (2)	0.091 (3)	0.007 (2)	0.057 (3)	−0.008 (2)
C1S	0.116 (7)	0.074 (5)	0.045 (4)	0.001 (5)	0.023 (4)	0.008 (4)
C2S	0.094 (6)	0.059 (4)	0.080 (5)	0.030 (4)	0.051 (5)	0.024 (4)

### Geometric parameters (Å, °)

**Table:**

Co1—N4	2.123 (4)	C17—C18	1.349 (8)
Co1—N2	2.129 (4)	C17—H17	0.9300
Co1—N6	2.129 (4)	C18—C19	1.428 (8)
Co1—N1	2.131 (3)	C18—H18	0.9300
Co1—N5	2.133 (4)	C19—C20	1.407 (8)
Co1—N3	2.142 (4)	C19—C23	1.413 (6)
N1—C1	1.331 (6)	C20—C21	1.357 (8)
N1—C12	1.353 (6)	C20—H20	0.9300
N2—C10	1.331 (6)	C21—C22	1.398 (7)
N2—C11	1.355 (6)	C21—H21	0.9300
N3—C13	1.327 (6)	C22—H22	0.9300
N3—C24	1.355 (6)	C23—C24	1.444 (7)
N4—C22	1.332 (6)	C25—C26	1.406 (7)
N4—C23	1.351 (6)	C25—H25	0.9300
N5—C25	1.334 (6)	C26—C27	1.362 (9)
N5—C36	1.366 (6)	C26—H26	0.9300
N6—C34	1.331 (6)	C27—C28	1.401 (8)
N6—C35	1.356 (6)	C27—H27	0.9300
C1—C2	1.398 (6)	C28—C36	1.400 (6)
C1—H1	0.9300	C28—C29	1.441 (8)
C2—C3	1.361 (7)	C29—C30	1.342 (8)
C2—H2	0.9300	C29—H29	0.9300
C3—C4	1.400 (7)	C30—C31	1.428 (7)
C3—H3	0.9300	C30—H30	0.9300
C4—C12	1.401 (6)	C31—C32	1.396 (7)
C4—C5	1.432 (7)	C31—C35	1.409 (6)
C5—C6	1.346 (8)	C32—C33	1.373 (8)
C5—H5	0.9300	C32—H32	0.9300
C6—C7	1.432 (7)	C33—C34	1.392 (7)
C6—H6	0.9300	C33—H33	0.9300
C7—C11	1.399 (6)	C34—H34	0.9300
C7—C8	1.413 (7)	C35—C36	1.429 (6)
C8—C9	1.359 (7)	B1—F1	1.344 (7)
C8—H8	0.9300	B1—F2	1.351 (7)
C9—C10	1.388 (7)	B1—F3	1.376 (7)
C9—H9	0.9300	B1—F4	1.426 (8)
C10—H10	0.9300	B2—F8	1.333 (8)
C11—C12	1.451 (6)	B2—F6	1.346 (7)
C13—C14	1.405 (7)	B2—F7	1.360 (7)
C13—H13	0.9300	B2—F5	1.468 (9)
C14—C15	1.353 (8)	C1S—C2S	1.438 (12)
C14—H14	0.9300	C1S—H1S1	0.9600
C15—C16	1.398 (8)	C1S—H1S2	0.9600
C15—H15	0.9300	C1S—H1S3	0.9600
C16—C24	1.405 (6)	C2S—N1S	1.139 (11)
C16—C17	1.440 (7)		
			
N4—Co1—N2	96.35 (14)	C24—C16—C17	118.5 (5)
N4—Co1—N6	94.96 (14)	C18—C17—C16	121.5 (5)
N2—Co1—N6	94.71 (14)	C18—C17—H17	119.3
N4—Co1—N1	170.19 (14)	C16—C17—H17	119.3
N2—Co1—N1	78.55 (14)	C17—C18—C19	121.4 (5)
N6—Co1—N1	93.82 (13)	C17—C18—H18	119.3
N4—Co1—N5	92.75 (13)	C19—C18—H18	119.3
N2—Co1—N5	168.89 (14)	C20—C19—C23	117.1 (5)
N6—Co1—N5	78.11 (14)	C20—C19—C18	124.0 (5)
N1—Co1—N5	93.34 (14)	C23—C19—C18	118.9 (5)
N4—Co1—N3	78.43 (14)	C21—C20—C19	119.6 (4)
N2—Co1—N3	91.24 (14)	C21—C20—H20	120.2
N6—Co1—N3	171.57 (13)	C19—C20—H20	120.2
N1—Co1—N3	93.21 (13)	C20—C21—C22	120.0 (5)
N5—Co1—N3	96.84 (14)	C20—C21—H21	120.0
C1—N1—C12	117.8 (4)	C22—C21—H21	120.0
C1—N1—Co1	128.9 (3)	N4—C22—C21	122.1 (5)
C12—N1—Co1	113.2 (3)	N4—C22—H22	118.9
C10—N2—C11	117.9 (4)	C21—C22—H22	118.9
C10—N2—Co1	128.8 (3)	N4—C23—C19	122.6 (4)
C11—N2—Co1	113.3 (3)	N4—C23—C24	117.7 (4)
C13—N3—C24	118.1 (4)	C19—C23—C24	119.7 (4)
C13—N3—Co1	129.0 (3)	N3—C24—C16	122.6 (4)
C24—N3—Co1	112.9 (3)	N3—C24—C23	117.5 (4)
C22—N4—C23	118.6 (4)	C16—C24—C23	119.9 (4)
C22—N4—Co1	127.9 (3)	N5—C25—C26	122.1 (5)
C23—N4—Co1	113.5 (3)	N5—C25—H25	119.0
C25—N5—C36	118.2 (4)	C26—C25—H25	119.0
C25—N5—Co1	128.6 (3)	C27—C26—C25	119.5 (5)
C36—N5—Co1	113.2 (3)	C27—C26—H26	120.2
C34—N6—C35	118.1 (4)	C25—C26—H26	120.2
C34—N6—Co1	128.1 (3)	C26—C27—C28	120.0 (5)
C35—N6—Co1	113.7 (3)	C26—C27—H27	120.0
N1—C1—C2	123.0 (4)	C28—C27—H27	120.0
N1—C1—H1	118.5	C36—C28—C27	117.4 (5)
C2—C1—H1	118.5	C36—C28—C29	119.1 (5)
C3—C2—C1	118.7 (4)	C27—C28—C29	123.6 (5)
C3—C2—H2	120.6	C30—C29—C28	120.9 (5)
C1—C2—H2	120.6	C30—C29—H29	119.5
C2—C3—C4	120.4 (4)	C28—C29—H29	119.5
C2—C3—H3	119.8	C29—C30—C31	121.4 (5)
C4—C3—H3	119.8	C29—C30—H30	119.3
C3—C4—C12	116.9 (4)	C31—C30—H30	119.3
C3—C4—C5	123.7 (4)	C32—C31—C35	117.5 (4)
C12—C4—C5	119.4 (4)	C32—C31—C30	123.6 (5)
C6—C5—C4	121.1 (4)	C35—C31—C30	118.8 (5)
C6—C5—H5	119.5	C33—C32—C31	119.8 (4)
C4—C5—H5	119.5	C33—C32—H32	120.1
C5—C6—C7	121.2 (4)	C31—C32—H32	120.1
C5—C6—H6	119.4	C32—C33—C34	119.0 (5)
C7—C6—H6	119.4	C32—C33—H33	120.5
C11—C7—C8	116.9 (4)	C34—C33—H33	120.5
C11—C7—C6	119.3 (4)	N6—C34—C33	123.1 (4)
C8—C7—C6	123.7 (4)	N6—C34—H34	118.4
C9—C8—C7	119.5 (4)	C33—C34—H34	118.4
C9—C8—H8	120.2	N6—C35—C31	122.5 (4)
C7—C8—H8	120.2	N6—C35—C36	117.5 (4)
C8—C9—C10	119.7 (5)	C31—C35—C36	120.0 (4)
C8—C9—H9	120.1	N5—C36—C28	122.8 (4)
C10—C9—H9	120.1	N5—C36—C35	117.5 (4)
N2—C10—C9	122.7 (4)	C28—C36—C35	119.7 (4)
N2—C10—H10	118.6	F1—B1—F2	112.6 (5)
C9—C10—H10	118.6	F1—B1—F3	111.9 (5)
N2—C11—C7	123.1 (4)	F2—B1—F3	113.7 (5)
N2—C11—C12	117.4 (4)	F1—B1—F4	105.9 (5)
C7—C11—C12	119.5 (4)	F2—B1—F4	105.7 (5)
N1—C12—C4	123.2 (4)	F3—B1—F4	106.3 (4)
N1—C12—C11	117.4 (4)	F8—B2—F6	116.8 (6)
C4—C12—C11	119.4 (4)	F8—B2—F7	113.9 (5)
N3—C13—C14	122.3 (5)	F6—B2—F7	111.6 (5)
N3—C13—H13	118.8	F8—B2—F5	105.2 (5)
C14—C13—H13	118.8	F6—B2—F5	104.2 (5)
C15—C14—C13	119.9 (5)	F7—B2—F5	103.5 (6)
C15—C14—H14	120.1	C2S—C1S—H1S1	109.5
C13—C14—H14	120.1	C2S—C1S—H1S2	109.5
C14—C15—C16	119.3 (5)	H1S1—C1S—H1S2	109.5
C14—C15—H15	120.4	C2S—C1S—H1S3	109.5
C16—C15—H15	120.4	H1S1—C1S—H1S3	109.5
C15—C16—C24	117.8 (4)	H1S2—C1S—H1S3	109.5
C15—C16—C17	123.6 (5)	N1S—C2S—C1S	178.8 (10)
